# Multimass Three-Dimensional Velocity Map Imaging from Surfaces

**DOI:** 10.1021/acs.jpclett.5c02672

**Published:** 2025-11-04

**Authors:** Yifeng Jia, Felicia M. Green, Kieran Cheung, Maria Elena Castellani, Mark Brouard

**Affiliations:** † The Department of Chemistry, 6396 University of Oxford, The Chemistry Research Laboratory, 12 Mansfield Road, Oxford OX1 3TA, United Kingdom; ‡ Rosalind Franklin Institute, Harwell Campus, Didcot OX11 0QX, United Kingdom

## Abstract

This paper presents a surface scattering study employing a multimass ion imaging method, in which the sample is mounted on the repeller plate of the ion optical system and ions generated at the surface are velocity mapped along the surface normal. Measurement of the ion position and arrival time at the detector using a fast imaging camera allows the direct measurement of the half-Newton spheres of multiple ions and thus the determination of the full three-dimensional velocity distributions of the species of interest. The method is illustrated by measuring the velocity distributions of multiple ions generated in secondary ion mass spectrometry (SIMS) experiments following impact by a kilo-electronvolt C_60_^+^ primary ion beam. The implications of the results for the dynamics of secondary ion formation are discussed. The three-dimensional velocity map imaging technique developed here should be applicable to a range of surface dynamical measurements in which ions are generated or extracted in a pulsed-like fashion to enable time-of-flight detection.

## Graphical abstract

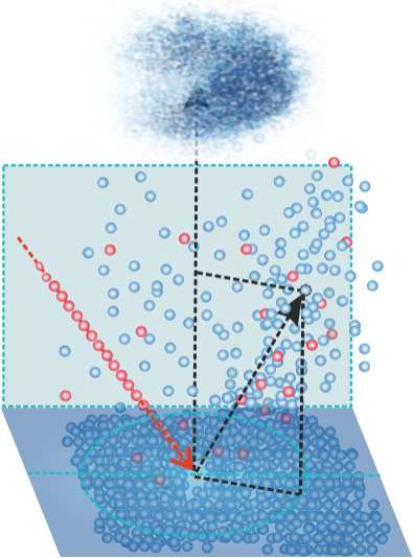

Velocity map^1^ ion imaging^2^ (VMI) is a well-established technique for determining the velocity distributions of ions generated by laser-induced methods in the gas phase. The method has been applied widely, particularly to study molecular photodissociation and inelastic and reactive scattering.^3^ Ion detection in VMI generally employs multichannel plates (MCPs) coupled with a phosphor screen and a charge-coupled device (CCD) camera. A variety of constraints on the latter means that VMI is most commonly used in a crushed or projected imaging mode in which the component of the ion velocity along the time-of-flight (ToF) axis is unresolved.^4^ To obtain more precise measurements with higher velocity resolution, slice ion imaging methods have been developed,^5–7^ in which the ion Newton sphere is expanded along the ToF direction to enable a central slice to be obtained, for example, by pulsing the MCP detector or the intensified CCD camera employed. In principle, slice imaging ion optics allow for the recording of the full three-dimensional (3D) Newton sphere of ions, provided the data rate and timing precision of the detector are sufficiently fast. For that reason, 3D VMI usually requires MCPs to be coupled with phosphor screens and fast complementary metal oxide (CMOS) detectors, such as the PImMS^8–11^ and TimePix-based cameras,^12–17^ with delay line detectors,^18,19^ or using fast MCP readout.^20^ Pulsed extraction methods can also be employed, for example, postextraction inversion slice imaging, to stretch the ion clouds still further, thereby increasing the number of slices achieved with moderate timing requirements.^11,21^

In the past decade, VMI has shifted focus from solely gas-phase measurements to surface (photo)­reactions^22–29^ and surface scattering.^30–33^ Because of the limited time resolution of conventional VMI detection systems, surface scattering measurements typically involve ion extraction in a direction parallel to the plane of the surface,^30,32^ so that the scattering angle from the surface normal can be more readily determined. In the present work, we show how 3D VMI allows the extraction of multiple ions along the surface normal using a simple ion optical arrangement in which the sample is directly mounted on the repeller plate of the instrument. While some previous studies, particularly those of surface photochemistry, have employed a similar geometry,^22,23,25–27^ namely, ion extraction along the surface normal, full 3D velocity information in such cases either has not been possible due to insufficient time resolution of the detector^25^ or has been derived from the ion signal obtained as a function of the time delay between photoexcitation at the surface and ionization by a probe laser, i.e., in a somewhat analogous manner to slice imaging.^22,23,26,27^ The multimass 3D VMI approach used here, in which the half-Newton spheres of ions are measured directly at the detector, is particularly advantageous in applications in which it is of interest to interrogate multiple species of different masses simultaneously, as in the present application.

In these proof-of-principle experiments, we use a sample comprising a thin film of an organic dye electrosprayed onto an indium tin oxide (ITO) cover slide, as previously employed in imaging mass spectrometry measurements.^34–37^ We use a modified secondary ion mass spectrometry (SIMS) instrument,^37^ coupled with 3D VMI using a TimePix3-based camera,^16^ to record 3D velocity distributions of multiple metallic and organic secondary ions generated at a surface following sputtering with a kilo-electronvolt (keV) C_60_^+^ primary ion beam.

While previous experimental measurements of the velocity distributions of secondary ions generated in SIMS have been pivotal in advancing our understanding of the sputtering process,^38–45^ particularly for monatomic primary ions on metals,^46–49^ less is known about the behavior of cluster ions impacting organic surfaces. A variety of molecular dynamics and experimental techniques, generally employing ToF mass spectrometry, have been used to investigate cluster ion beams (including C_60_^+^) contacting metallic surfaces^41,50–52^ and organic substrates.^39,42,45,52–54^ The primary beam incident angle and beam energy influence the velocity distributions of secondary ions ejected from the surface, and several mechanisms have been proposed to rationalize both computational^52,53^ and experimental^42,55–57^ findings. Experiments of the type described here should provide further insight into the mechanism of secondary ion production.

The 3D VMI technique employed here is illustrated schematically in Figure 1. Following C_60_^+^ primary ion beam sputtering of the sample (see Figure 1a), secondary ions were extracted along the surface normal using a simple ion optical arrangement (Figure 1b). By adjusting the voltage ratios among the repeller, extractor, and lens, ion trajectories could be focused according to either their spatial position on the surface (Figure 1c) or their velocity at their instant of their birth (Figure 1d,e). The ability to switch readily from spatial to velocity mapping of the secondary ions generated at the surface, in principle on alternate primary beam pulses in a multimass fashion, opens the possibility of being able to characterize the dynamics in specific regions of the surface. Note that it is possible to focus or defocus the primary beam on alternate pulses, with a focused beam used to perform VMI on a well-defined region identified with a defocused beam in spatial mapping mode. In spatial mapping mode, one could identify regions of interest; then one could switch to velocity map imaging using a more focused primary ion beam to record the velocity distributions for secondary ions specifically from those identified regions of interest.

**Figure 1:**
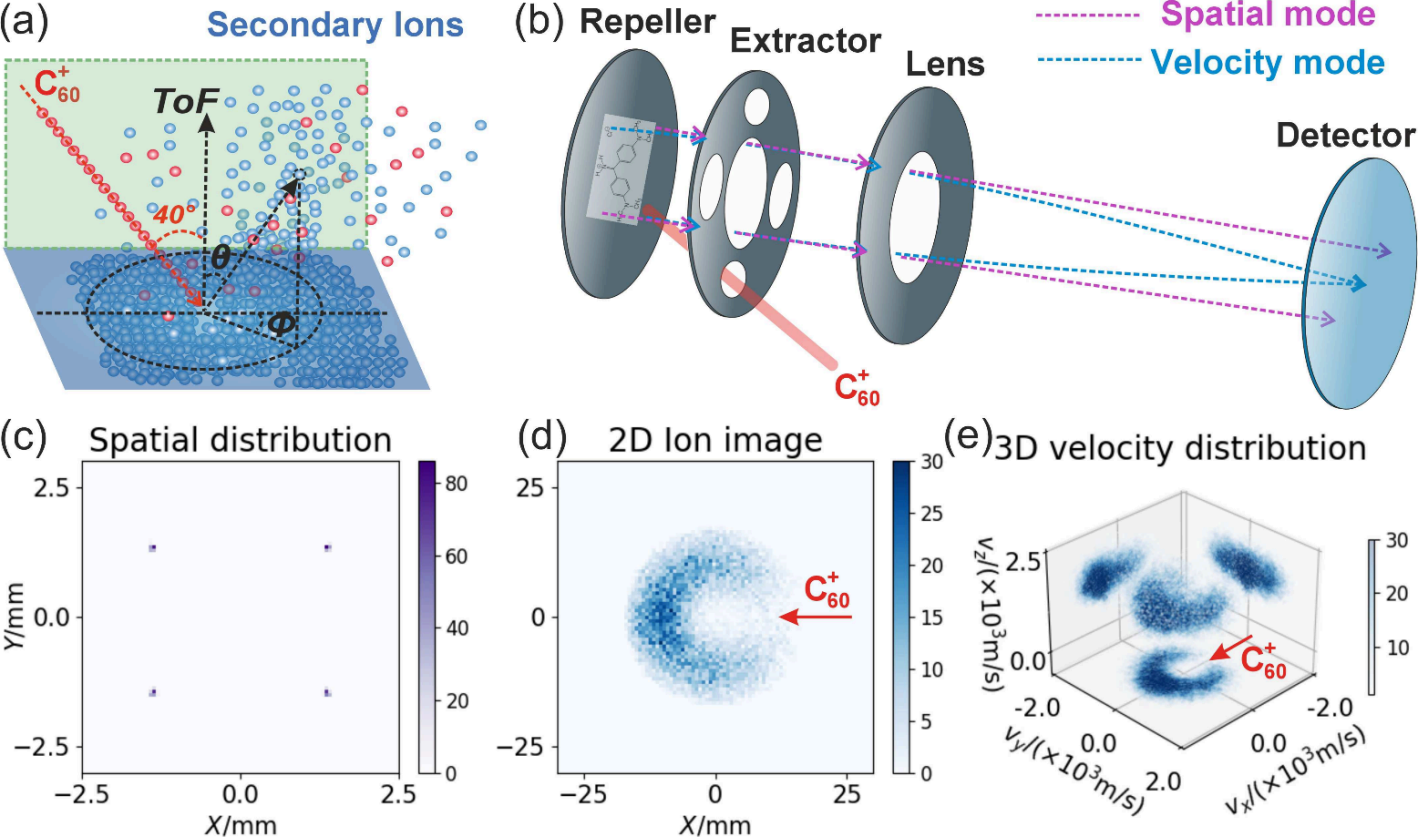
(a) Collisional process in SIMS. A 20 or 40 keV C_60_^+^ ion beam is used as the primary ion source. After bombarding the target surface, the sample’s chemical components undergo fragmentation and ionization. (b) After ionization, the secondary ions are accelerated and focused using a conventional VMI ion lens setup. By adjusting the ion optical voltages on the electrodes, the ion image can be switched between either spatial mapping mode (purple dashed line) or velocity mapping mode (blue dashed line). (c) Simulated spatial ion image showing four focused points on the detector, corresponding to the spatially mapped initial positions of the ions. (d) Simulated “crushed” 2D velocity map ion image showing events on the detector, corresponding to the mapped initial velocities of the ions. (e) Simulated ion velocity distributions as observed in a 3D velocity map image. The ions have an assumed mass of 269 Da, and their kinetic energy ranges from 0 to 1.2 eV.

To measure accurate 3D velocity distributions, high ion timing precision is required to ensure that the velocity components along *x*, *y*, and *z* are sampled as equally as possible. Here, we define the surface to lie in the *x–y* plane with the ToF direction defining the *z*-axis. In this work, ions were detected using a standard dual-MCP/P47 imaging detector coupled either to a conventional CCD camera or to a TimePix3-based camera.^16^ The TimePix3-based camera records ion positions with a ToF timing precision of 1.56 ns, so this represents ∼60 time slices for every 100 ns portion of the ToF spectrum. For the organic ion at *m*/*z* 269 (see below), the full width of the ToF peak could be stretched out to around 180 ns, with a full width at half-maximum (fwhm) of ∼75 ns, corresponding to 115 and 48 time bins, respectively. This number of time bins, which is directly related to the resolution of the velocity component along the *z*-axis, should be compared with the number of pixels in the TimePix3 array, 256 × 256, of which at most 128 characterize the radius of the image, i.e., the speed along an axis in the *x–y* plane.

An important aspect of the 3D VMI experiments is the velocity focusing and calibration of the instrument, further details of which are provided in the Experimental Methods and Supporting Information.

An example ToF mass spectrum, obtained at a C_60_^+^ primary beam energy of 20 keV using the TimePix3-based camera, is shown in Figure 2a for an Auramine O laser dye sample (see Experimental Methods). 3D velocity distributions for this sample were determined for the Na^+^, K^+^, and [M-Cl^–^]^+^ (the parent ion of Auramine O) ions, with these peaks identified in the mass spectrum. Magnified ToF spectra are also displayed in the bottom panels of Figure 2a. The bottom row of panels (Figure 2b–d) displays the two-dimensional (2D) crushed velocity map images. It is notable that the organic ions exhibit a very different 2D-projected velocity map ion image compared with metallic ions, reflecting differences in the sputtering and ionization processes responsible for their formation. Further examples of 2D-projected velocity map images, for both the parent and fragment ions, are shown in Figure S7. These reveal that fragment ions with masses closer to that of the Auramine O parent ion show similar, anisotropic 2D-projected velocity map images, while lower-mass fragments display almost isotropic 2D-projected images, more similar to those of the metallic ions.

**Figure 2:**
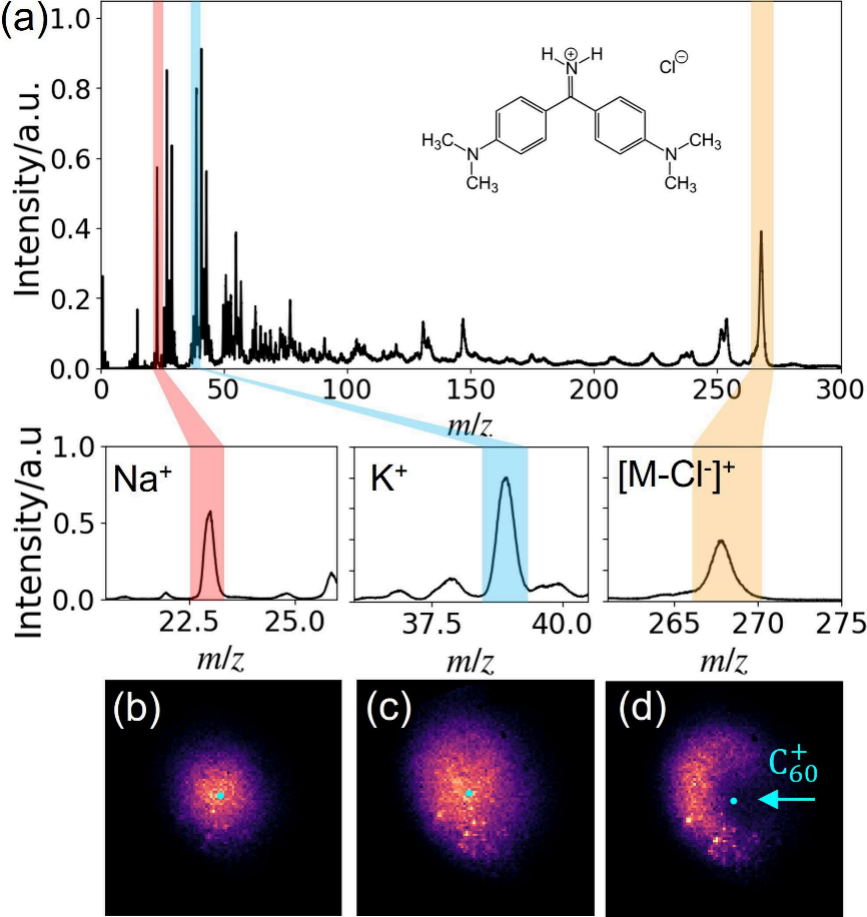
(a) ToF spectrum (top) of Auramine O (structure shown in the panel) measured with the TimePix3 camera at a C_60_^+^ primary beam energy of 20 keV. Expanded view (middle) of the ToF spectra for three secondary ions, Na^+^ (left), K^+^ (middle), and [M-Cl^–^]^+^ (right), obtained under velocity mapping conditions. (b–d) Corresponding 2D velocity map images for the Na^+^, K^+^, and [M-Cl^–^]^+^ ions, respectively. The center of the velocity map images is shown by the cyan dot. The experiments were performed with repeller, extractor, and ion lens voltage settings of 3.0, 2.79, and 2.4 kV, respectively.

Figure 3 shows the full 3D velocity and angular distributions derived from the 3D velocity map images for the Na^+^, K^+^, and [M-Cl^–^]^+^ ions, bombarded by a 20 keV C_60_^+^ primary beam. For an incident C_60_^+^ injection angle of 40° with respect to the surface normal, the organic parent ions exhibit an azimuthal angle distribution, *P*(ϕ), peaking around ϕ = 0°, but with a broad distribution of ∼155°. More notably, the organic [M-Cl^–^]^+^ ion distribution is seen to peak away from the surface normal, with θ ∼ 20°, while the metallic ions, Na^+^ and K^+^, peak close to the surface normal at θ = 0°.

**Figure 3:**
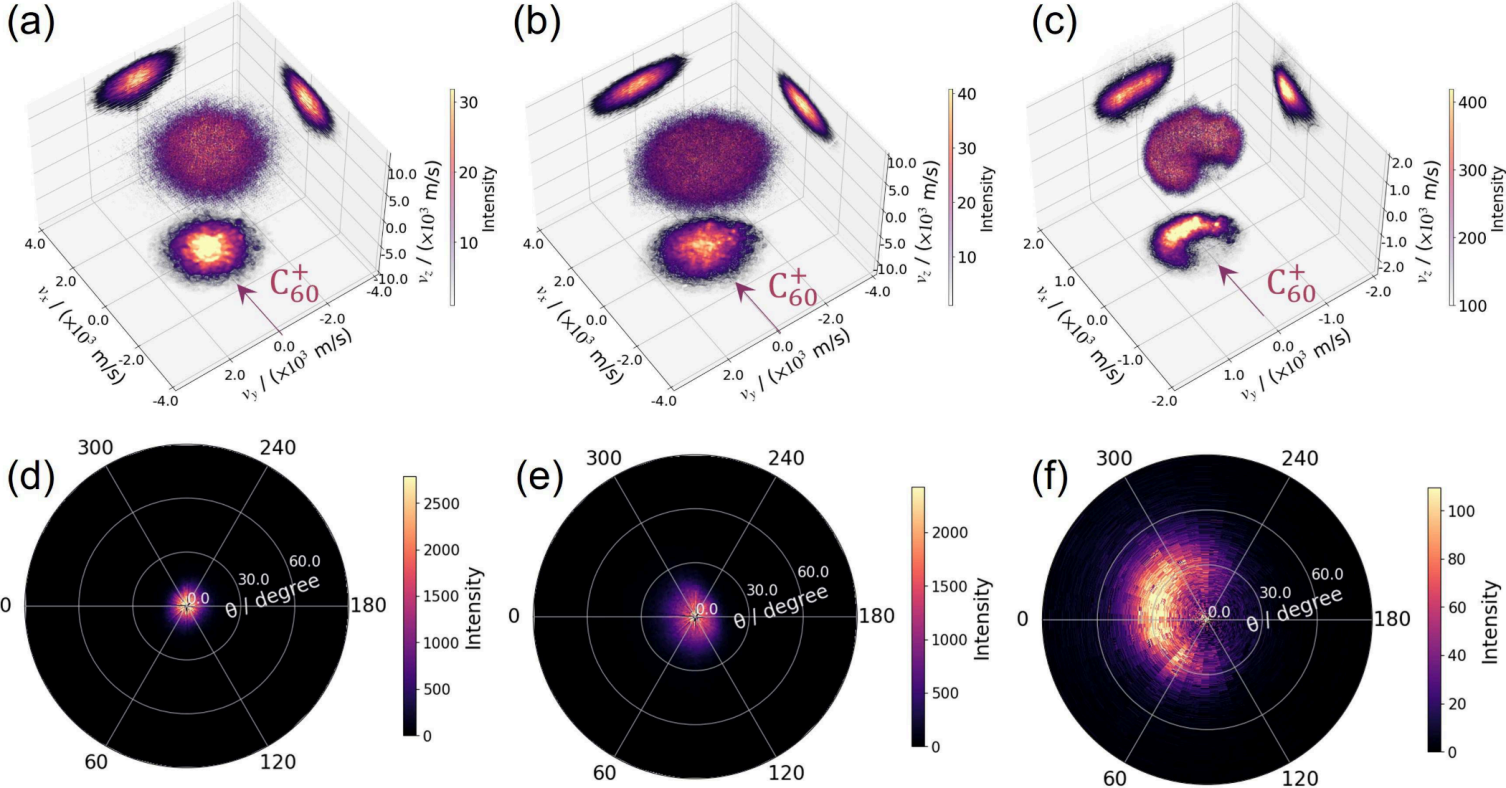
(a–c) Comparisons of the 3D velocity distributions obtained for the Na^+^, K^+^, and [M-Cl^–^]^+^ ions, respectively, following C_60_^+^ sputtering of an Auramine O sample at 20 keV. The experiments were performed with repeller, extractor, and ion lens voltage settings of 3.0, 2.79, and 2.4 kV, respectively. (d–f) Comparisons of the associated *P*(θ, ϕ) angular distributions for Na^+^, K^+^, and [M-Cl^–^]^+^, respectively.

The velocity distribution data can be used to derive the polar angle, momentum, and kinetic energy distributions for the Na^+^, K^+^, and [M-Cl^–^]^+^ ions, as shown in Figure 4, again employing a 20 keV C_60_^+^ primary beam. For the polar angle and momentum distributions, a Gaussian fit function is applied, while a Maxwell–Boltzmann fit function is used for the kinetic energy distribution. The means and fwhm of these distributions are listed in Table 1. The data confirm that for Na^+^ and K^+^ the polar angle distributions, *P*(θ), peak around θ = 0°, while the [M-Cl^–^]^+^ organic ions have a broader distribution with a mean around θ = 22°. Note also that the momentum distributions for the two metallic ions are very similar and have considerably lower mean momenta than the organic species.

**Figure 4:**
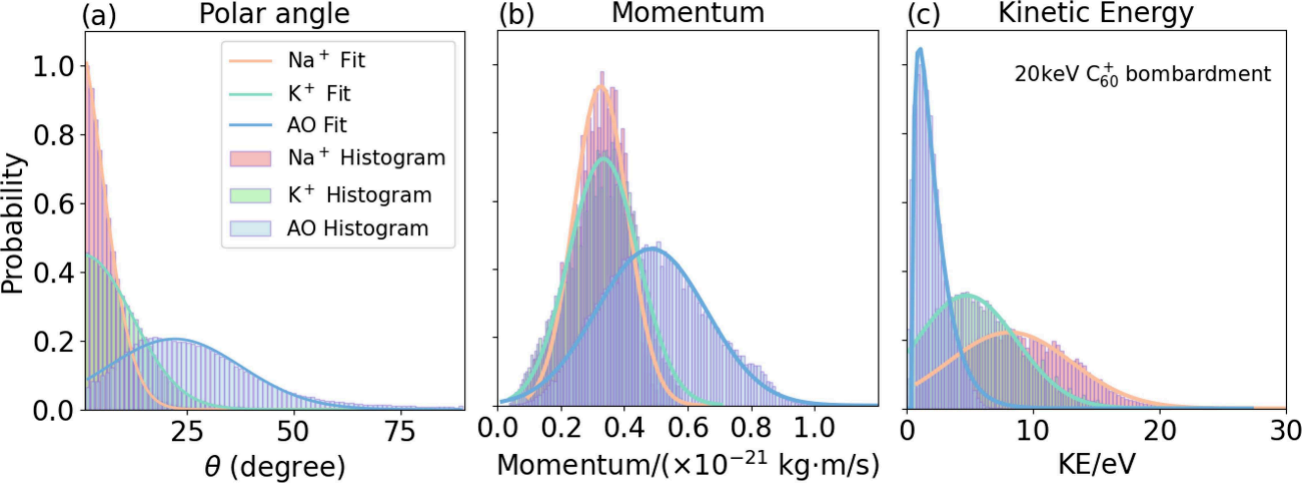
(a–c) Polar angular, momentum, and kinetic energy distributions, respectively, for the Na^+^ (red), K^+^ (green), and [M-Cl^–^]^+^ (blue) ions following C_60_^+^ sputtering of an Auramine O (AO) sample at 20 keV.

**Table 1:** Key Properties of the Polar Angle, Momentum, and Kinetic Energy Distributions for Selected Ion Species Arising from Sputtering with 20 keV (top three rows) and 40 keV (bottom three rows) C_60_^+^ Primary Ions

ion species	mean θ (deg)	fwhm θ (deg)	mean momentum (×10^–22^ kg m^–1^ s^–1^)	fwhm momentum (×10^–22^ kg m^–1^ s^–1^)	mean KE (eV)	fwhm KE (eV)
20 keV						
Na	0.56 ± 0.16	17.14 ± 0.17	3.33 ± 0.02	2.01 ± 0.04	8.29 ± 0.08	10.85 ± 0.15
K	0.96 ± 0.19	26.39 ± 0.27	3.35 ± 0.01	2.60 ± 0.03	4.87 ± 0.06	8.35 ± 0.11
AO	22.12 ± 0.25	36.86 ± 0.67	4.90 ± 0.02	4.22 ± 0.04	1.51 ± 0.04	2.81 ± 0.07
40 keV						
Na	0.91 ± 0.10	14.51 ± 0.12	4.76 ± 0.01	0.90 ± 0.02	18.17 ± 0.98	7.00 ± 2.36
K	0.61 ± 0.19	20.59 ± 0.26	4.76 ± 0.01	1.92 ± 0.02	9.90 ± 0.98	8.67 ± 2.36
AO	17.05 ± 0.11	25.90 ± 0.28	7.06 ± 0.04	4.69 ± 0.07	3.25 ± 0.03	4.60 ± 0.06

To better understand the sputtering and ionization processes, we also measured the secondary ion velocity distributions with a 40 keV primary ion beam. To compare with the 20 keV data shown in Figure 2, the full 3D velocity distributions for the Na^+^, K^+^, and [M-Cl^–^]^+^ ions at both 20 and 40 keV primary ion beam energies are shown in Figure S8. Comparisons of the resulting polar angle, momentum, and kinetic energy distributions for each of these ions at both sputtering energies are shown in Figure 5, with the means and fwhm of these distributions also summarized in Table 1.

**Figure 5:**
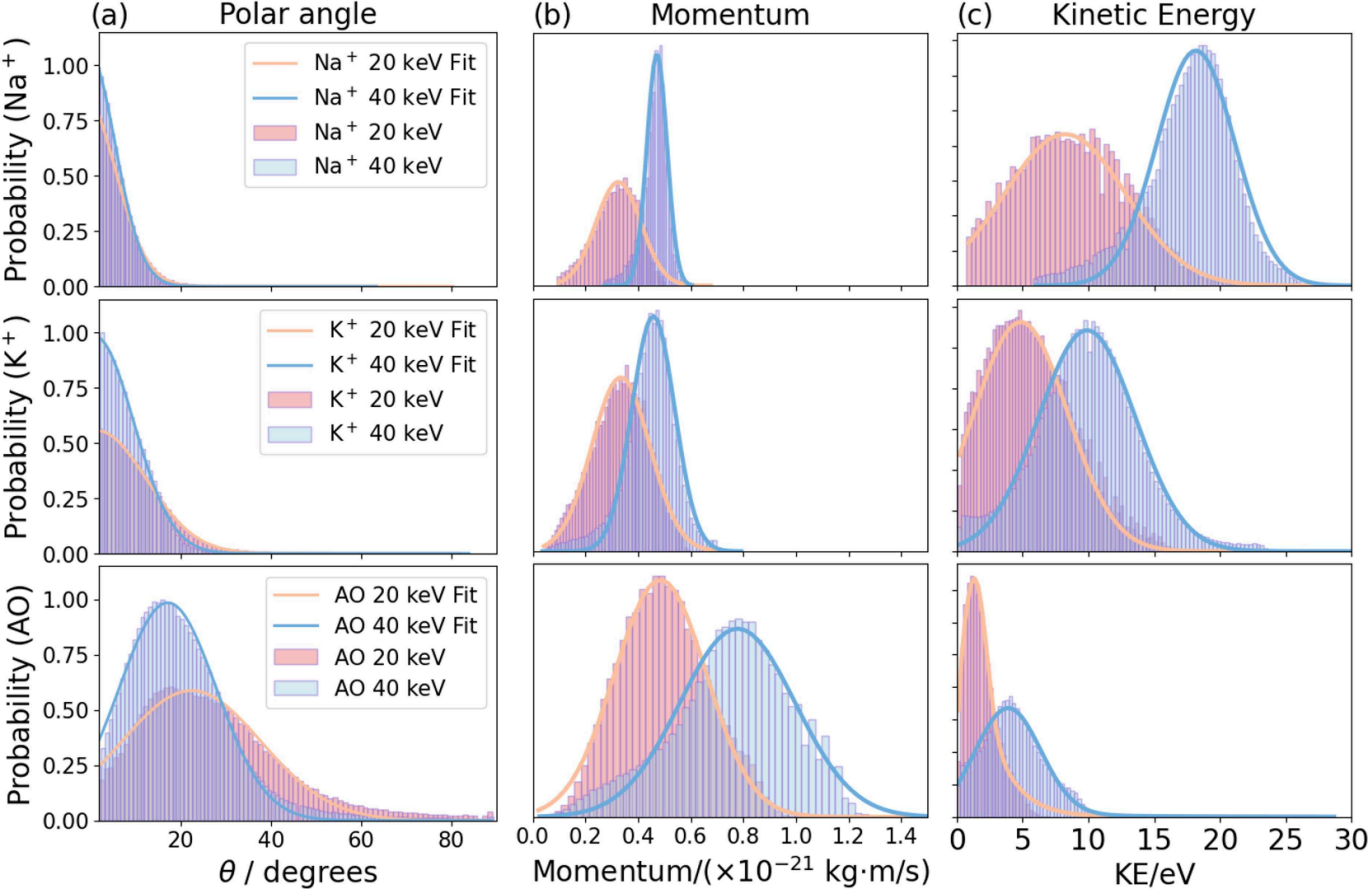
(a–c) Comparisons of the polar angular, momentum, and kinetic energy distributions, respectively, for the Na^+^ (top row), K^+^ (middle), and [M-Cl^–^]^+^ (bottom row) ions generated following C_60_^+^ sputtering of an Auramine O (AO) sample at 20 and 40 keV. In each plot, the effect of the primary ion beam energy (20 keV in red, 40 keV in blue) is compared.

The general pattern of behavior observed for the Na^+^, K^+^, and [M-Cl^–^]^+^ ions at a 20 keV primary beam energy is repeated at 40 keV (see Figure 5 and Table 1). The metallic ions are distributed very close to the surface normal, about which they almost have cylindrical symmetry. The organic ion, on the other hand, is scattering significantly away from the surface normal, with nonaxial symmetry. The momentum distributions for the two metallic ions are almost identical to each other at both sputtering energies, with significantly higher momentum carried away by the organic [M-Cl^–^]^+^ ion, suggesting that Na^+^ and K^+^ are formed by a similar ionization mechanism, while different dynamics operate for the organic ion. Note also that when the primary ion beam energy is doubled, the mean kinetic energies of the secondary ions approximately double, and the mean momentum of the secondary ions increases by a factor of $\sqrt{2}$ (see Figure 5).

A key factor in explaining the observed behavior is likely to be the location of the ions at their point of generation. Given that the surface of the sample is electrosprayed with the organic dye in question, it seems reasonable to suppose that the organic ions originate from the top surface layer of the sample. Na^+^ and K^+^ ions are ubiquitous contaminants and are very readily ionized via mass spectrometry. However, the fact that increasing the primary ion beam energy results in a much larger increase in Na^+^ and K^+^ signals compared to the organic ions suggests that the metallic ions are more concentrated deeper into the surface, closer to the ITO-coated substrate, as accessed by the higher-energy C_60_^+^ beam. Note that due to the manufacturing process of ITO slides, it would be unsurprising to find sodium and potassium ions present.^58,59^ Given that the organic layer is only ∼100 nm deep (see Experimental Methods), and that the typical penetration depth of a C_60_^+^ ion is between 20 and 50 nm,^60^ it seems likely that the higher-energy C_60_^+^ ions will release more Na^+^ and K^+^ ions, which will be more concentrated closer to the ITO surface. One would expect that the organic ions, originating from the surface, will show evidence of formation by a more direct sputtering process, leading to an anisotropic angular distribution and a relatively high momentum transfer from the primary ion compared with the more readily ionized metallic species, originating from regions closer to the ITO-coated substrate.

The momentum distributions of the metallic and organic ions support a model in which incoming C_60_^+^ ions transfer significantly more momentum to the surface organic ions than to the underlying metallic ions. As noted above, the momentum of both the metallic and organic secondary ions is enhanced by a factor of about $\sqrt{2}$ when the primary ion beam energy is doubled, which means that a constant fraction of the incoming C_60_^+^ ion momentum is transferred to the secondary ions during the sputtering process, this fraction being rather smaller for the metallic ions than for the surface organic ions. Consistent with this increase in secondary ion momentum with an increase in primary ion beam momentum, the mean kinetic energies of three secondary ions roughly double with a doubling of the primary beam kinetic energy. This kind of momentum transfer process agrees with previous research by Kelner and Markey^61^ and Magee.^62^

The polar angular distributions provide further detailed dynamical information, with a higher primary ion beam energy leading to somewhat smaller θ angles and sharper polar angular distributions, as shown in Figure 5a. This effect is more significant for organic than for metallic ions (see Table 1). This could be due to the increased injection depth of the primary ions at higher energy. A deeper injection means that the edge of the crater and the depth of the ion scattering can influence the secondary ion trajectories, pushing them toward the normal direction and thus reducing θ angles and sharpening angular distributions. It is also noteworthy that the momentum distributions for the organic ion become somewhat broader with a higher primary ion beam energy, while metallic ions show sharper distributions (see Figure 5b and Table 1). This observation may also be related to the penetration depth of the primary ion, and the subsequent secondary ion scattering dynamics.

As noted in the introduction, the sputtering process in SIMS is relatively well understood for metallic ions sputtered by monatomic primary ions, where a “linear collision cascade” leads to the ejection of atoms from the surface^63–65^ and to beam-induced evaporation.^65^ However, for cluster primary ions, it has been shown that a linear model no longer holds, and additional mechanisms need to be considered in order to account for the observed sputtering. These mechanisms rely on the collective motion caused by multiple low-energy atoms hitting the surface at the same position simultaneously, leading to a collective transfer of kinetic energy and subsequent thermal desorption.^65^ Sometimes this has been subdivided into two sputtering mechanisms, those of the ions central to the collision area that yield higher-kinetic energy secondary ions^42,55,56^ and those desorbed from the edges of the collision region that lead to secondary ions with lower kinetic energies.^66^ Several different names have been used to describe these two mechanisms, but here we will consider them as linear and thermal, respectively. The linear collision cascade mechanism usually produces sputtered ions after a number of collisions have taken place and hence produces secondary ions with uniform angular distributions about the surface normal and with higher kinetic energies than the thermal mechanism. Such behavior is similar to that observed for the underlying metallic ions, Na^+^ and K^+^, studied here. Thermal sputtering occurs immediately at the sample surface and is usually anisotropic; the ballistic collision process is strongly impacted by the primary ion injection angle; hence, the angular distributions tend to reflect the asymmetry of the primary ion motion. This behavior is similar to the scattering observed here for the organic surface ions.

The dynamic information presented here points to the location and nature of the secondary ion as being of key importance, with surface organic ions showing more direct sputtering and higher momentum releases than the metallic ions. The latter, Na^+^ and K^+^, which are likely to arise from regions below the surface closer to the ITO substrate, display more isotropic sputtering patterns with lower momenta than for the surface organic ions. This behavior could reflect both the multiple collisions experienced by the incoming C_60_^+^ primary ion beam and the increased likelihood of multiple collisions experienced by secondary ions originating from deeper in the surface organic dye layer.

In summary, we have demonstrated the measurement of 3D velocity distributions of multiple ions generated in a surface scattering experiment using a simple VMI ion optical system coupled to a TimePix3-based camera. We directly measure the full 3D velocity distributions, or half-Newton spheres, of multiple ions following their extraction along the surface normal, which is enabled by the high time resolution in the ToF coordinate afforded by the TimePix3-based camera. The methods developed here should be applicable to other surface photoinitiated processes, including laser desorption ionization (LDI) and matrix-assisted laser desorption ionization (MALDI), and to surface scattering experiments, provided that the detected ions are produced in a pulsed-like fashion to allow ToF mass spectrometry analysis. Note that these processes could be studied in either positive or negative ion mode. Specifically, in the case of scattering, we envisage that multiple species of interest could be detected using universal femtosecond near-infrared nonresonant ionization^29,67^ coupled with the 3D VMI methods described here. Note that the time delay between the photoinitiation or scattering event could be varied or scanned to ensure detection of the full velocity range of the species of interest.

We demonstrated the approach by measuring the 3D velocity distributions of multiple secondary ions generated in a SIMS sputtering process. Specifically, we use a C_60_^+^ primary ion beam impacting on a meshed Auramine O dye sample. The data reported provide insights into the effect that the primary ion beam energy has on secondary ion angular and momentum distributions. Furthermore, we find significant differences in the 3D velocity distributions (both in angle and momentum) of the organic and metallic ions, which can be attributed to distinct sputtering and ionization processes, depending on the depth within the surface at which the secondary ions are formed and extracted. This work underscores the complexity of the SIMS sputtering and ionization process in determining the momentum distributions of secondary ions, suggesting that further simulations would be helpful.

## Experimental Methods

The experimental arrangement is shown schematically in panels a and b of Figure 1. The mass spectrometer employed is a modified SIMS instrument designed for microscope mode imaging mass spectrometry, which has been described in detail previously.^37^ The experiments employed a 20 or 40 keV C_60_^+^ primary ion beam, which impacted with the surface of interest 40° to the surface normal. A simple ion optical setup, consisting of a repeller plate, on which the sample is mounted, the extractor, and an ion lens assembly, was employed to map the secondary ions in spatial and velocity mapping modes, as shown in Figure 1b. Mapping of spatial position at the surface or velocity was controlled by adjusting the voltage ratio among the repeller, extractor, and lens. The detector system (Photonis 39042PS-P47) consisted of two 40 mm imaging-quality Chevron microchannel plates (MCPs) and a fiber-optic-coupled phosphor screen (P47 phosphor) mounted on a 6 in. vacuum flange. The MCPs were operated with the front of the first plate set to −840 V, the back of the first plate connected to the front of the second plate (at ground), and the back of the second plate set to 840 V. The phosphor screen was set between 4.0 and 4.6 kV, and the emission was detected and recorded for subsequent analysis using either an intensified CCD camera, a TimePix3-based camera,^16^ or a photomultiplier tube (PMT). The VMI data were recorded using the TimePix3-based camera at a C_60_^+^ repetition rate of 100 Hz for around 15 min, with a primary ion beam current of 850–900 pA. Under these conditions, we expect around 550 C_60_^+^ ions to collide with the surface per ion-gun pulse. For the 20 keV experiments, roughly 100 secondary ions were detected per C_60_^+^ pulse, which increased to around 300 ions/pulse at 40 keV.

Samples of different laser dyes (Rhodamine 640 and Auramine O) were electrosprayed, using a custom-built electrosprayer, onto a standard ITO-coated cover slide, which was placed on the repeller plate of the ion optical system (Figure 1b). The samples were also sprayed onto a nickel mesh, with the resulting square ion image pattern formed in spatial mapping mode used to calibrate the ion image. Atomic force microscopy measurements, supported by calculations based on electrospray flow rate and solution concentration, indicate a dye sample depth of ∼90 ± 30 nm. The 3D VMI data shown in the main text used the Auramine O mass peak at *m*/*z* 269, as well as those for the Na^+^ and K^+^ ions. Additional calibration data shown in the Supporting Information used the Rhodamine 640 mass peak at *m*/*z* 491.

Ion trajectories were simulated using SIMION 2020.^68^ For the data shown in Figure 1, ions were initialized at four distinct positions, but with the same velocity distribution. In the spatial mapping mode, the detected signal is shown in Figure 1c, while the simulated velocity-mapped 2D-crushed image and 3D-scattered velocity distributions are shown in panels d and e, respectively, of Figure 1. In the spatial map image, the signal corresponds to the target molecule positions, which appear as four distinct circular points. In contrast, VMI is relatively unaffected by the spatial position of the targeted molecule, but rather by its initial velocity. Thus, in VMI mode, ions with different spatial positions, but identical velocities, will still be focused onto the same point on the detector. Simulations performed assuming that ions are generated in an area of 100 μm on the surface, as in the experiments, indicated that they are velocity mapped onto the detector with a velocity resolution that is limited by the pixel number in the camera.

The TimPix3-based camera provides ToF data in the form (*x_i_*, *y*_*i*_, *t*_*i*_), where (*x*_*i*_, *y*_*i*_) are the position coordinates (pixel numbers) in the detector plane and *t*_*i*_ is the arrival time (time stamp) of ion *i* at the detector. For each ion event, these data must be transformed into (*v*_*x*,*i*_, *v*_*y*,*i*_, *v*_*z*,*i*_), where the *z*-axis is taken as the ToF axis and +*x* defines the direction of the C_60_^+^ primary beam. In most VMI experiments, velocity calibration is achieved by photodissociation of a gas-phase molecule producing fragments with a known well-defined velocity distribution. In the present work, we used an alternative procedure, involving a combination of ion trajectory simulation coupled with calibration of those simulations against experimental spatial mapping and time-focusing data. Further experimental details are provided in the Supporting Information.

## Supplementary Material



## Notes

Spatial and velocity focus (section S1), image centering, *x*_0_, *y*_0_ (section S2), determining the ToF origin, *t*_0_ (section S3), and 3D velocity calibration and distributions (section S4) (PDF)
