# Supplementary material for: Multimass Three-Dimensional Velocity Map Imaging from Surfaces
Source: J Phys Chem Lett. 2025 Nov 4;16(45):11762–9. doi: 10.1021/acs.jpclett.5c02672 (PMC12621235; doi:10.1021/acs.jpclett.5c02672)
Supplement: Supplementary file 1 [file jz5c02672_si_001.pdf]

# Supporting information for: Multi-mass three-dimensional velocity map imaging from surfaces

Yifeng Jia,[†] Felicia M. Green,[‡] Kieran Cheung,[†] Maria Elena Castellani,[†] and Mark Brouard[†]

[†] *The Chemistry Research Laboratory, Department of Chemistry, University of Oxford, Oxford OX1 3TA, United Kingdom*

[‡] *The Rosalind Franklin Institute, Harwell Campus, OX11 0QX, United Kingdom*

## Table of contents

S1 Spatial and velocity focus

S2 Image centering, $x_0, y_0$

S3 Determining the ToF origin, $t_0$

S4 3D velocity calibration and distributions

# S1 Spatial and velocity focus

In a gas phase experiment the velocity focus and calibration in a velocity map imaging (VMI) experiment is readily accomplished using molecular photodissociation to generate a photofragment born with a known, well-defined velocity. Although, in principle, the same technique could be used in the present experiments, this was not possible on the instrument employed. Alternative methods were therefore developed, based on a combination of ion trajectory simulation using SIMION [1] and calibration experiments.

Unlike the case with velocity in VMI, it is relatively straightforward in the secondary ion mass spectrometry (SIMS) instrument to determine the spatial focus and calibration. This was achieved by adjusting the ratio of the extractor and repeller voltages until a sharp image of a grid pattern of known dimension was obtained, as illustrated in Fig. S1. It was established that the experimental voltage settings aligned very well with results from ion trajectory simulations, thus providing confidence in the use of the simulations as a guide in use of the surface VMI experiments.

In the simulations, the spatial focus was quantified using the spatial resolution, where a smaller resolution corresponds to better spatial focus. To determine the spatial resolution, three groups of ions are initialized at three distinct positions on the repeller, separated by a distance, $d_1$. When these ions reach the detector, their final positions are recorded, with a new separation distance on the detector, $d_2$. The ratio $M = d_2/d_1$ determines the magnification of the instrument. Each ion group forms a spot on the detector with a spatial spread characterized by a standard deviation, which can be used to calculate the full-width-at-half-maximum (FWHM) of the signal intensity on the detector. The spatial resolution is then given as the FWHM of the spot on the detector divided the magnification, FWHM$/M$. The calculated spatial resolution as a function of the voltage ratios between the extractor and repeller, $V_{\mathrm{E}}/V_{\mathrm{R}}$, and lens and repeller, $V_{\mathrm{L}}/V_{\mathrm{R}}$, is shown in Fig. S1(a), with the dark blue region corresponding to the best spatial resolution, which indicates a spatial resolution below $5\,\mu$m. The voltage ratios for the optimum spatial resolution is plotted as blue dots in Fig. S1(c).

The velocity focus in the simulations was determined using the Pearson correlation coefficients, $\rho_{\mathrm{ToF}}$ and $\rho_{x/y}$. Note that the surface normal lies along the ToF direction, defined here as the $z$-axis, with the surface lying in the $(x, y)$ plane. $\rho_{\mathrm{ToF}}$ refers to the correlation coefficient between

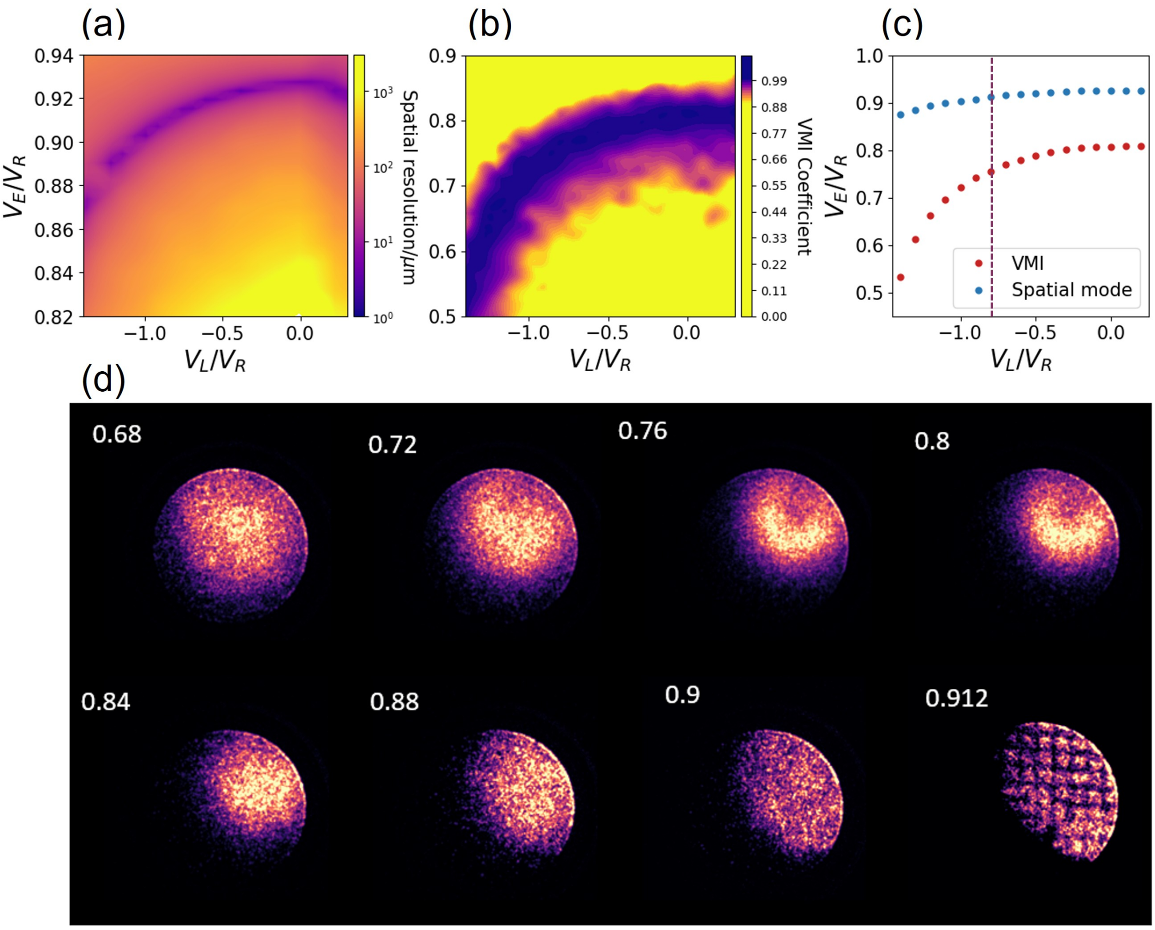


Figure S1: Simulation of the optimisation of the lens-to-repeller, $V_L/V_R$, and extractor-to-repeller, $V_E/V_R$, voltage ratios, with dark blue indicating improved (a) spatial resolution and (b) improved velocity mapping as indicated by the Pearson coefficient, $\rho_x$. (Note the non-linear colour scales used in panels (a) and (b).) Panel (c): A comparison of the optimized voltage ratios, $V_E/V_R$, for spatial (blue dots) and velocity (red dots) map imaging. Panel (d): Experimental ion images of a Rhodamine 640 sample (using the peak at $m/z = 491$) at a repeller voltage of 10 kV, a lens voltage of $-8$ kV (as indicated by the dashed vertical line in panel (c)), and with varying extractor/repeller ratios. A defocussed $C_{60}^+$ beam produces a spatial image of the sample grid pattern at a ratio of 0.912. The simulation predicts an optimum velocity focus ratio of 0.76, consistent with the experimental ion images shown.

the measured time-of-flight (ToF) $t_{\mathrm{ToF}}$ and the velocity along the ToF axis ($v_{\mathrm{ToF}}$):

$$\rho_{\mathrm{ToF}} = \frac{\mathrm{cov}(t_{\mathrm{ToF}}, v_{\mathrm{ToF}})}{\sigma_{t_{\mathrm{ToF}}} \sigma_{v_{\mathrm{ToF}}}}, \tag{1}$$

where $\mathrm{cov}(\cdots)$ refers to the covariance between $t_{\mathrm{ToF}}$ and $v_{\mathrm{ToF}}$, while $\sigma_{t_{\mathrm{ToF}}}$ and $\sigma_{v_{\mathrm{ToF}}}$ represent the standard deviations of $t_{\mathrm{ToF}}$ and $v_{\mathrm{ToF}}$, respectively. Similarly, the velocity focus along $x$ in the

image plane was determined using the Pearson correlation coefficient

$$\rho_x = \frac{\mathrm{cov}(x, v_x)}{\sigma_x\, \sigma_{v_x}}\,. \tag{2}$$

where $\sigma_x$ and $\sigma_{v_x}$ represent the standard deviations of position on the detector $x$ and the speed along the detector plane $v_x$, and similarly for the $y$ coordinate in the image plane. To achieve a good velocity focus, both $\rho_{\mathrm{ToF}}$ and $\rho_{x/y}$ should be used, although in practice, the $\rho_{\mathrm{ToF}}$ coefficient exhibits a significantly larger tolerance than the $\rho_{x/y}$ coefficient, indicating that velocity focusing along the TOF axis is more easily achieved than in the detector plane.

A Pearson coefficient close to +1 or −1 indicates a strong linear relationship, while values near 0 suggest a weak correlation. Because the position coordinate, $x$ or $y$, and the corresponding velocity component, $v_x$ or $v_y$, are direct correlated, the velocity mapping condition is better determined when the coefficient $\rho_x$ or $\rho_y$ is close to +1, as demonstrated in the blue stripe in Fig. S1(b). For each lens to repeller voltage ratio ($V_{\mathrm{L}}/V_{\mathrm{R}}$), the optimal extractor/repeller voltage ratio ($V_{\mathrm{E}}/V_{\mathrm{R}}$) are shown in Fig. S1(c), where the optimal spatial mapping settings (blue dots) are compared to the velocity mapping settings (red dots). These data provide a practical guide when it comes to the experimental setup.

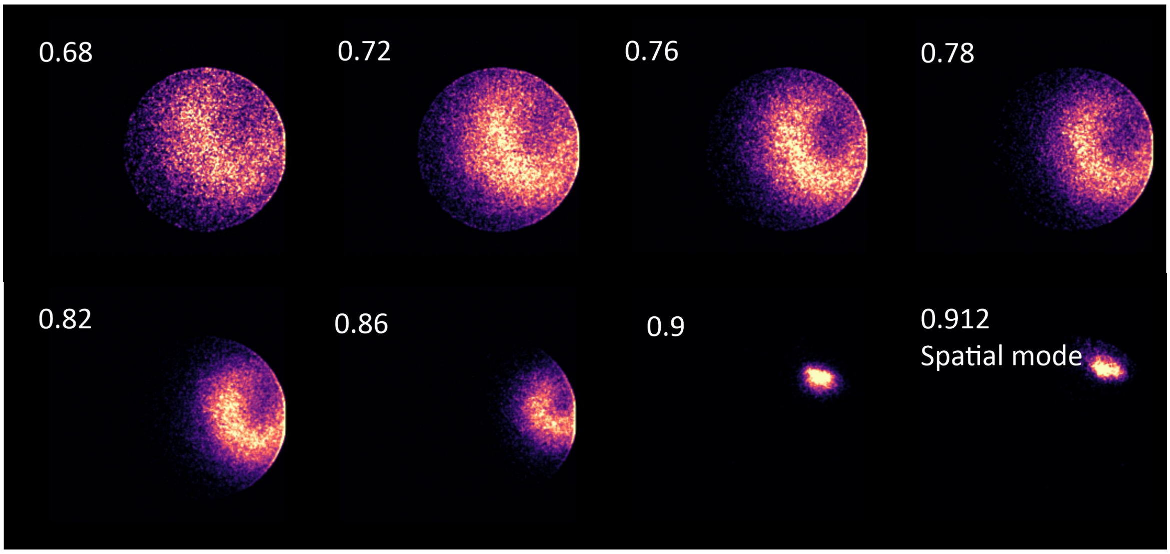


Figure S2: As for Fig. S1(d), but showing ion images for the Rhodamine 640 sample (using the peak at $m/z = 491$) obtained with a $\sim 100\,\mu$m focused primary ion beam with varying $V_{\mathrm{E}}/V_{\mathrm{R}}$ voltage ratios, with $V_{\mathrm{R}} = 10\,$kV, $V_{\mathrm{L}} = -8\,$kV.

To obtain the velocity focus in the image plane, a defocussed 40 keV $C_{60}^{+}$ beam was used to

bombard a meshed grid with a pitch size of 140 $\mu$m electrosprayed with Rhodamine 640 dye. The repeller voltage was set at 10 kV, while the $V_L/V_R$ ratio was fixed at $-0.8$ to achieve a reasonable magnification. This value is labelled with the dashed vertical line in Fig. S1(c). As shown in Fig. S1(d), as the $V_E/V_R$ ratio was scanned from around 0.6 to 0.95, the spatial mapping condition is clearly achieved when $V_E/V_R$ was 0.912. Under these conditions, a distinct grid pattern is detected, as expected from the simulation in Fig. S1(c). As $V_E/V_R$ is decreased, the image becomes increasingly blurry and eventually transitions into a half-ring pattern when $V_E/V_R$ is 0.76. This voltage ratio aligns well with the suggested velocity focus ratio of 0.758 predicted from the simulations. Further reduction in the ratio causes the pattern to become more diffuse and eventually disappear, indicating the loss of velocity focus, as seen from the data shown in Fig. S1(d).

For a defocussed primary ion beam, as used in Fig. S1(d), the velocity focus will be somewhat compromised due to the finite size of the ionization source region on the surface, which leads to differences in flight path of the secondary ions created further away from the center of the extraction optics. To address this, subsequent experiments were conducted using a focused $C_{60}^+$ primary beam, with a spot size of approximately 100 $\mu$m. As shown in Fig. S2, the secondary ion image in spatial mode is now focused into a spot rather than a large circle. In Fig. S2, ion images are shown when the ratio voltage scan experiment was repeated with the focused $C_{60}^+$ primary beam. The tolerance of the velocity focus to differing $V_E/V_R$ ratios was improved significantly compared to the defocussed primary ion beam experiments, but, importantly, the optimal ratio setting was found to remain close to 0.76, as expected based on the ion trajectory simulations.

# S2 Image centering, $x_0, y_0$

To determine absolute velocity values, the centre of the velocity map image, $x_0, y_0$, and the origin in time along the ToF axis, $t_0$, first need to be established. Effectively, these parameters define the laboratory origin in the 3D image, as indicated schematically in Fig. S3(a).

The origin of the ion image corresponds to the position on the detector that a secondary ion would hit if it had no angular component to its velocity (as illustrated in Fig. S3(a)), *i.e.* when $\theta = 0°$ and the ion flies directly along the ToF $z$-axis. This defines the velocity origin in the $(x, y)$ plane of the image. When the primary ion strikes the surface, the secondary ion is

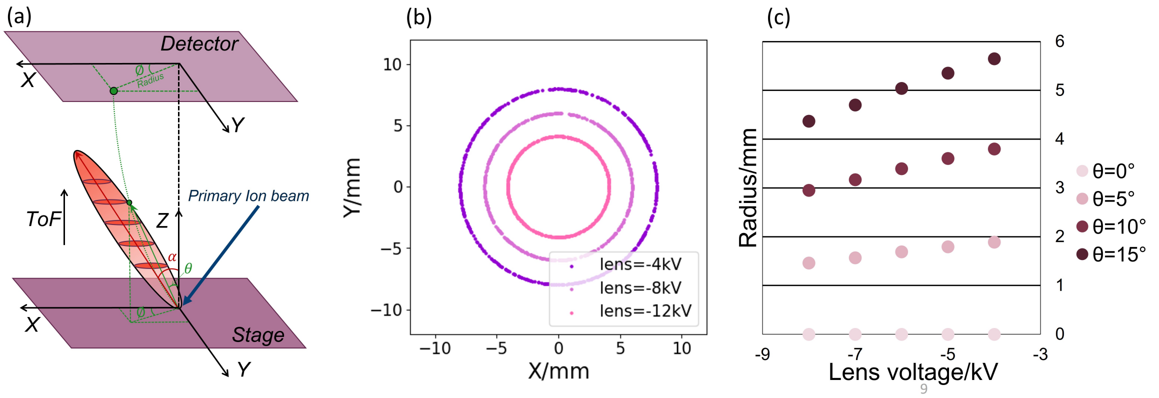


Figure S3: Panel (a): A schematic illustrating the transformation strategy from camera parameters to ion velocity. A single ion is depicted as a green dot, with its azimuth angle $\phi$ and polar angle $\theta$, recorded by the camera as an array of position $(x, y)$ and time-of-arrival. The ion cluster or plume is shown as an orange plume, with an average polar angle $\alpha$. Panel (b): Scattering plot of the secondary ions velocity distribution, recorded with the same repeller potential and lens voltages of $-4$, $-8$, and $-12\,\mathrm{kV}$, with $\theta = 30°$. The plot shows the velocity distribution of ions at different angles. Panel (c): The ion positions on the detector relative to the central axis and the lens voltage for ions with different scattering angles.

typically ejected with a non-zero $\theta$ angle, with larger $\theta$ values, at a given speed, mapped further away from the centre of the ion image. As shown in the main text, the 3D velocity distribution, and hence also the 2D projected image will not necessarily be cylindrically symmetric, making it difficult to identify the central point directly as the centroid of the image. To address this issue, we introduced a signal magnification procedure to help define the image centre.

By adjusting the lens and extractor voltages, the magnification of the ion signal changes. In the simulation shown in Fig. S3(b), 269 $m/z$ ions with a single kinetic energy of 1.7 eV and a cone angle distribution of $\theta = 30°$ were used. The repeller voltage was set to 10 kV, and the lens voltage was varied from $-4$, $-8$, to $-12\,\mathrm{kV}$. For a specific $\theta$, increasing the lens voltage is seen to lead to a decrease in signal magnification. This magnification effect is quantified and shown in Fig. S3(c). Simulations reveal that as the secondary ion polar angle $\theta$ increases, the ion signal on the detector becomes more sensitive to changes in the lens potential. However, there is a single point — the origin of the image, at which $\theta = 0°$ — that remains unaffected by the lens voltage. Thus, by varying the lens potential, thereby varying the image magnification, it is possible to isolate the origin of the image in the $xy$ plane. The signal position is unaffected by the lens potential only when $\theta = 0°$; for larger values of $\theta$, the sensitivity to lens voltage becomes

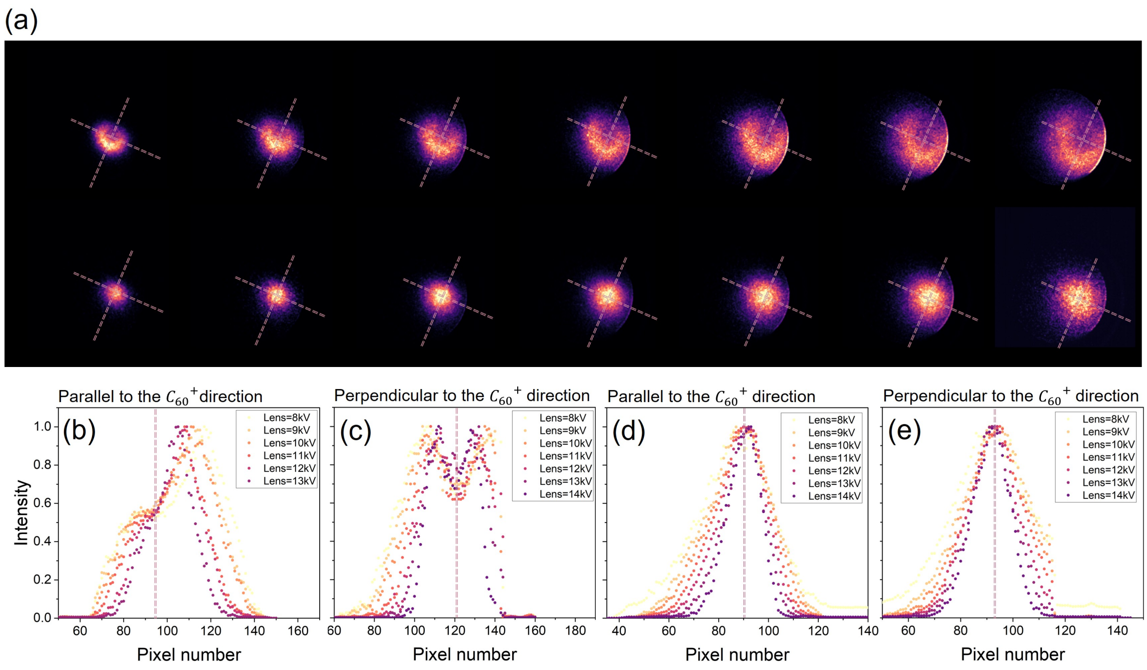


Figure S4: Panel (a): Ion images of Rhodamine 640 (top panel - peak at $m/z = 491$) and $Na^{+}$ (bottom panel) with varying lens voltages, ranging from 14 kV to 4 kV, decreasing in steps of 2 kV. Panels (b) and (c): Signal intensity profiles for the Rhodamine 640 images, measured parallel and perpendicular to the $C_{60}^{+}$ primary ion beam direction, respectively. Panels (d) and (e): Signal intensity profiles for the $Na^{+}$ images, measured parallel and perpendicular to the $C_{60}^{+}$ primary ion beam direction, respectively.

more pronounced.

Fig. S4(a) shows images at varying magnification from Rhodamine 640 (top row) and $Na^{+}$ (bottom row) ions. In these experiments, the repeller voltage was set to 10 kV, and the lens voltage was varied from −8 kV to −14 kV in 1.0 kV steps, with all the ion images captured using an intensified CCD camera. Superimposed on the images are two dashed lines corresponding to motion parallel and perpendicular to the $C_{60}^{+}$ primary beam direction. Fig. S4(b) and (c) show the normalized signal intensities along these two orthogonal directions for the Rhodamine 640 ions, whilst Fig. S4(d) and (e) show the same for the $Na^{+}$ ions. As the signal magnification increases, the width of the intensity distribution broadens, while the central point of the image stays fixed, as indicated by the purple dashed lines in the figures. In this way, the procedure enables the centre of the VMI image to be determined.

## S3 Determining the ToF origin, $t_0$

Because velocity is inherently 3D, calibrating the velocity along the ToF axis is also important, as it influences the derived ion speed and angular distributions. Therefore, the definition of the origin of the Tof axis, $t_0$, is also essential. $t_0$ defines the arrival time of an ion born at the surface with zero speed and mapped along the surface normal parallel to the time-of-flight axis. To find $t_0$ in the experiments, we used a calibration method based on the post-extraction differential acceleration [2–4] and post-extraction inversion slice imaging [5] techniques previously employed to focus or defocus ToF peaks by applying a pulse to the extractor plate of the ion optics. In this work, we made use of the fact that by applying a specific pulsed voltage to the extractor and varying the pulse trigger time, a specific mass peak will be time-focussed at two trigger times. This arises because at the time of pulsing the extractor the ion cloud may either have passed the extractor plate or may not have reached it yet, and therefore the ions are either accelerated or decelerated along the ToF, respectively, depending on their location. We label the ToF arrival times of these two time-focussed peaks $t_1$ and $t_2$. Because $t_1$ and $t_2$ can be measured precisely in the experiment, with the help of simulation we can use those values to calculate $t_0$. Note that $t_0$ is the ToF arrival time without a trigger pulse applied.

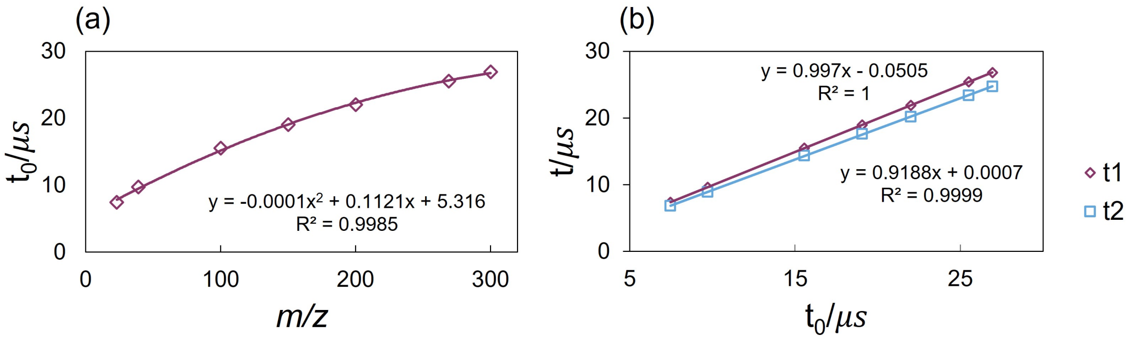


Figure S5: Panel (a): The relationship between different ion masses and their corresponding $t_0$ values. Panel (b): The relationship between the two time-focused times ($t_1$ and $t_1$) and the $t_0$. The data in both panels was obtained through SIMION simulation of the experiment.

In the SIMION simulations (see Fig. S5), we first tested different ion masses and recorded their ToF when the velocity along the ToF axis was $0\,\mathrm{m\,s^{-1}}$. A quadratic relationship between $t_0$ and mass was observed, as shown in Fig. S5(a), as expected. By varying the pulse trigger time, the values for $t_1$ and $t_2$ were also determined. From the simulated data shown in Fig. S5(b), we

can see that these times, $t_1$ and $t_2$, exhibit a linear relationship to $t_0$, which can then be used to calibrate the $t_0$ by matching to experimental data.

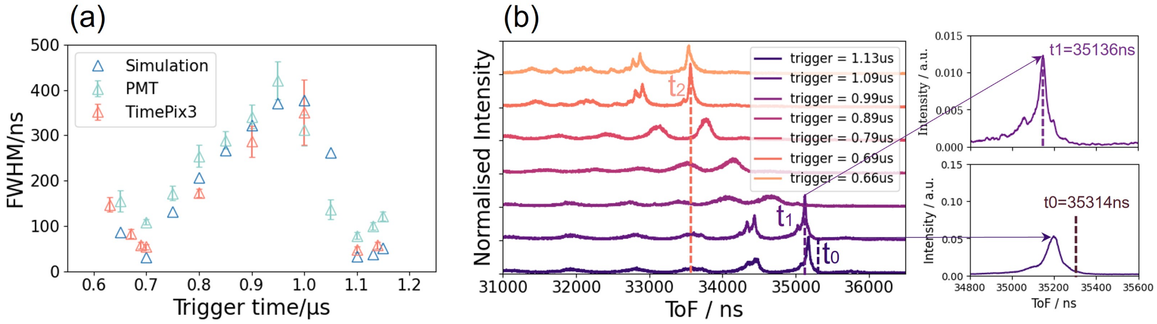


Figure S6: Panel (a): A comparison between the FWHM of the ToF mass peaks obtained using the experimental post extraction calibration data obtained from TimePix3-based camera and a PMT detector, and the corresponding simulation results. This comparison verifies the accuracy of the experimental setup. Panel (b): Time-of-flight (ToF) peak focusing by varying the pulse trigger time. The two time-focused ToF arrival times, $t_1$ and $t_2$, are indicated, showing how the pulse triggers affect the ToF distribution. The sample employed was Auramine O.

In the experiments (see Fig. S6), the initial voltages of the repeller, extractor, and lens were set to 3 kV, 2.79 kV, and 2.4 kV, respectively. At the trigger time (indicated in panel Fig. S6(b)), the extractor voltage was increased to 3.79 kV, with a pulse response time of less than 100 ns. The time-focus was determined by observing the variation in FWHM as a function of the trigger delay time. The latter was varied in 10 ns increments, and the FWHM of the targeted ion peak was measured using both a photomultiplier (PMT) and the TimePix3-based camera. The two arrival times, $t_1$ and $t_2$, that give focused ToF peaks were found to align very well with those from the ion trajectory simulations (Fig. S6(a)). Note that the FWHM of the ToF peaks collected by the PMT are slightly larger than those observed with TimePix3-based camera and the simulation (see Fig. S6(a)). Because the TimePix3 pixels are triggered on the leading edge of the emission from the phosphor screen, TimePix3-based camera records somewhat smaller FWHMs than the PMT data, which is limited by the response time of the P47 phosphor screen employed on the MCP detector. Representative experimental ToF data are shown in Fig. S6(b), illustrating the two time-focussing conditions that are achieved varying the trigger delay, leading to the time-focused mass peaks at $t_1$ and $t_2$. Their relationship with the origin time, $t_0$, obtained without a trigger pulse applied is also indicated in the right hand panel.

## S4 3D velocity calibration and distributions

Once the image centre, $x_0, y_0$, and the ToF origin, $t_0$, are defined, the transformation strategy is outlined in Fig. S3(a). The 3D velocity distribution is obtained using the transforation Eqs. (3)-(5), which allow the mapping of the measured ion event in the image, $x_i, y_i, t_i$, into laboratory frame velocity, $v_{x,i}, v_{y,i}, v_{z,i}$. The specific scaling between detector position, $(x, y)$ and time-of-flight $t_{\text{tof}}$ and the 3D velocity were obtained from the SIMION simulations, suitably calibrated using the experimental data as discussed above. In the simulation, ions were initialized with a spatial distribution of 100 $\mu$m in diameter and, separately, a linear velocity distribution along both the TOF axis and the detector plane. Under the VMI conditions employed, SIMION was used to calculate the ToF of each ion and its impact position on the detector. This output information was then used to fit the calibration equations in both in-plane directions and along the ToF direction. Specifically in the case of the $Na^+$ ions under the experimental conditions described, this transformation leads to the calibration equations:

$$v_x = [-0.3056 \times (x - x_0) - 0.0221] \times 1000 \quad (\mathrm{m\,s^{-1}}) \tag{3}$$

$$v_y = [-0.3056 \times (y - y_0) - 0.0221] \times 1000 \quad (\mathrm{m\,s^{-1}}) \tag{4}$$

$$v_z = \left[-710.24 \times (t_{\text{ToF}} - t_0)^2 - 191.04 \times (t_{\text{ToF}} - t_0) + 0.008\right] \times 1000 \quad (\mathrm{m\,s^{-1}})\,, \tag{5}$$

where $x$ and $y$ are the ion positions on the detector in millimeters, and $t_{\text{ToF}}$ is measure in microseconds. Eq. (3) represents the velocity in the $x$-direction, while Eq. (4) corresponds to that along $y$-direction. The velocity along the $t_{\text{ToF}}$ direction is given by Eq. (5). $t_0$ defines the laboratory origin along the ToF axis, as discussed in Section S3, whilst $x_0$ and $y_0$ define the laboratory origin in the image plane, as discussed in Section S2. Similar equations were readily defined for ions of different mass.

Some noise is present in the simulation results used to define the calibration equations, mainly due to sampling a finite spot size of the primary ion beam. However, this noise is minimal, and we are able to achieve excellent VMI focusing with correlation coefficients exceeded 0.99. For instance, in the simulation of a 23 Da ion with a velocity of 600 $\mathrm{m\,s^{-1}}$ in the detector plane and a 100 $\mu$m spatial spread, in VMI mode the resulting detector position was at a radius of $1.970 \pm 0.012$ mm. Thus, the standard deviation in the velocity calibration is better than 1%, confirming the robustness of the velocity mapping simulations.

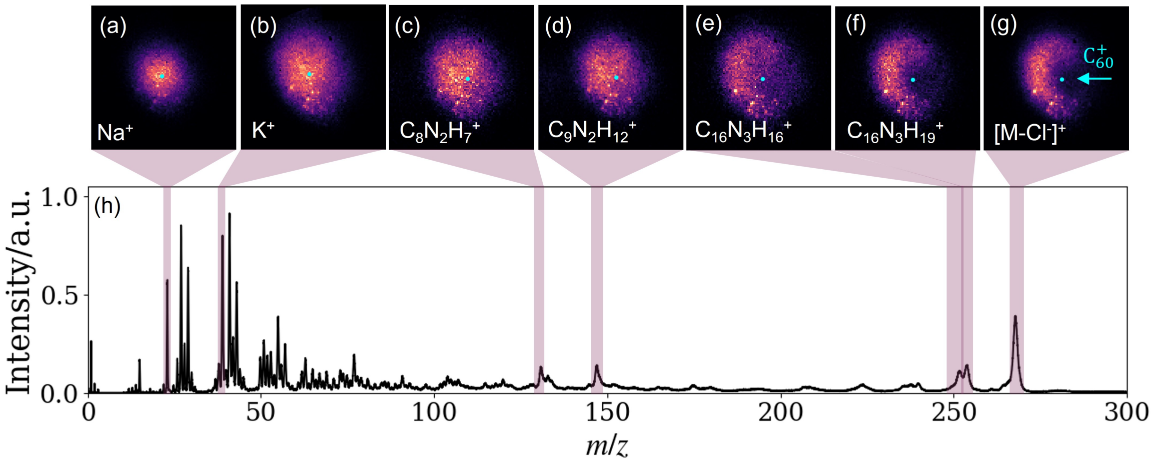


Figure S7: Top panels (a)-(g): Example 2D projected velocity map images for a range of ions, including $Na^+$, $K^+$, and $[M\text{-}Cl^-]^+$ (Auramine O (AO) parent) shown in the main text, together with some of the AO fragment ions. The centre of the velocity map images is shown by the cyan dot. The experiments were obtained with the repeller, extractor, and ion lens voltage settings of 3.0 kV, 2.79 kV and 2.4 kV, respectively. Bottom panel (h): Associated ToF mass spectrum of AO measured with the TimePix3 camera at a $C_{60}^+$ primary beam energy of 20 keV.

In the experiments described in the main text, an Auramine O dye was coated on a standard indium tin oxide (ITO) cover slide, and bombarded with $C_{60}^+$ primary ions at 20 keV and 40keV. Fig. S7 shows an example of the ToF spectrum of Auramine O dye obtained at the lower sputtering energy, together with a more complete set of 2D projected velocity map images for both the parent ions and some of its fragments, as well as those for $Na^+$ and $K^+$. It is interesting to observe that, as the degree of fragmentation of the parent increases, so the 2D projected images change from being highly anisotropic around the image centre, to more isotropic, with those for the smaller fragment ions closely resembling the 2D images of the metallic ions.

The full 3D velocity distributions of the $Na^+$, $K^+$, and $[M\text{-}Cl^-]^+$ ions were determined using the methods detailed in the preceding sections. We used both 20 keV and 40 keV $C_{60}^+$ primary beam energies to bombard the sample. The measured 3D velocity distributions of these three ions at both beam energies are shown in Fig. S8. While the primary ion beam energy does not significantly affect the shape of the velocity distribution, with metallic ions showing a consistent circular distribution and the Auramine O parent ions exhibiting a crescent-shaped distribution, the energy is seen to have a substantial effect on the ion speeds. Specifically, the velocity values in each dimension are amplified by approximately $\sqrt{2}$ factor when the primary ion beam energy is increased from 20 keV to 40 keV, as discussed further in the main text.

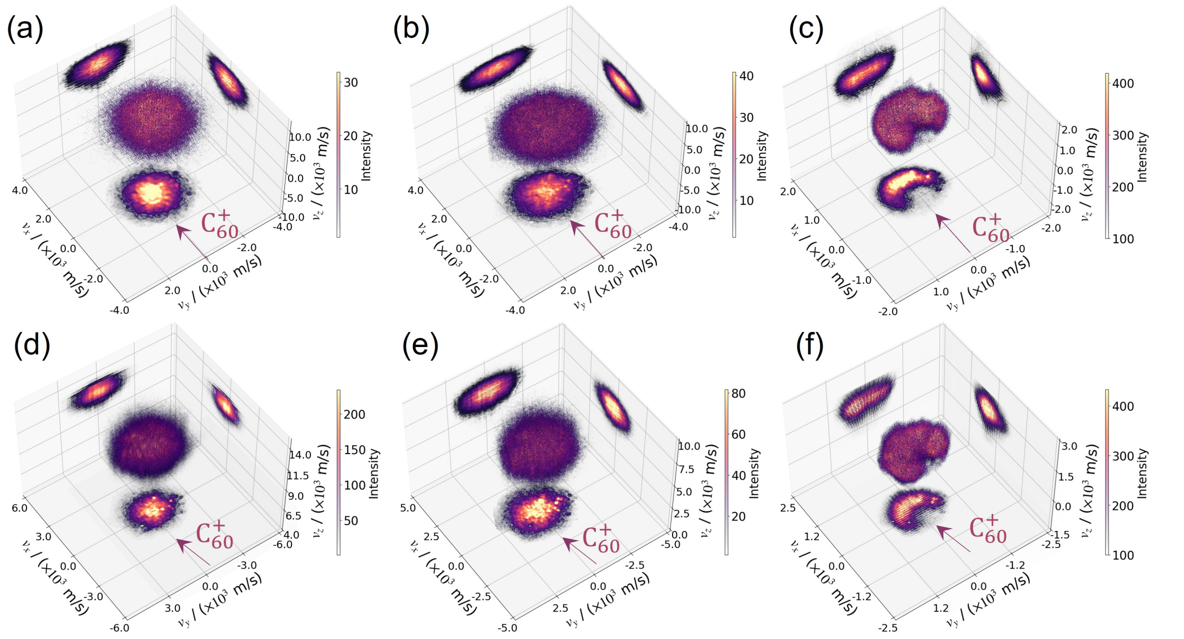


Figure S8: Panels (a)-(f): The 3D velocity distributions of various ions measured with 20 keV and 40 keV $C_{60}^{+}$ beams. The sample used was Auramine O dye. The various panels show the 3D velocity distributions for: (a) $Na^{+}$ with 20 keV $C_{60}^{+}$; (b) $K^{+}$ with 20 keV $C_{60}^{+}$; (c) $[M\text{-}Cl^{-}]^{+}$ with 20 keV $C_{60}^{+}$; (d) $Na^{+}$ with 40 keV $C_{60}^{+}$; (e) $K^{+}$ with 40 keV $C_{60}^{+}$, and (f) $[M\text{-}Cl^{-}]^{+}$ with 40 keV $C_{60}^{+}$.

# References


[1] D. Manura, *Adaptas Solutions, LLC (Adaptas)*, 2006-2020.

[2] J. J. Aoki, H. Hazama, and M. Toyoda, *J. Mass Spectrom. Soc. Jpn.*, 2011, **59**, 57.

[3] A. Guo, M. Burt, and M. Brouard, *Int. J. Mass Spectrom.*, 2018, **429**, 121–126.

[4] A. Guo, R. J. Burleigh, N. Smith, M. Brouard, and M. Burt, *J. Am. Soc. Mass Spectrom.*, 2020, **31**, 1903–1909.

[5] F. Allum, R. Mason, M. Burt, C. S. Slater, E. Squires, B. Winter, and M. Brouard, *Mol. Phys.*, 2021, **119**, e1842531.
